# The Healthy Eating Index 2020 components contributed unequally to systemic inflammatory biomarkers: A large national cross‐sectional study

**DOI:** 10.1002/fsn3.4347

**Published:** 2024-07-16

**Authors:** Hongguang Yang, Yao Liu, Zhenhe Huang, Guifang Deng

**Affiliations:** ^1^ Department of Clinical Nutrition Union Shenzhen Hospital of Huazhong University of Science and Technology Shenzhen China; ^2^ Department of Geriatric Medicine Union Shenzhen Hospital of Huazhong University of Science and Technology Shenzhen China

**Keywords:** added sugars, dietary pattern, Healthy Eating Index 2020 components, systemic inflammation, whole fruits, whole grains

## Abstract

The association of Healthy Eating Index 2020 (HEI‐2020), especially its components with systemic inflammatory biomarkers, has not been examined. The aim of this study was to investigate the relationship between HEI‐2020 and its components with systemic inflammatory biomarkers, and to provide a dietary pattern suggestion to combat systemic inflammation. Participants aged 20 years and older with complete information on two reliable dietary recalls, blood cell counts and demographic characteristics, were recruited from six NHANES cycles from 2007 to 2018. Weighted general linear methods showed that HEI‐2020 was negatively associated with systemic immune‐inflammation index (SII) and systemic inflammation response index (SIRI). Weighted quantile regression (WQS) models and quantile g‐computation (QGC) models supported the negative association between the mixed components and systemic inflammation. High intakes of whole fruits, whole grains, greens and beans, and seafood and plant proteins, along with a low intake of added sugars, were associated with reduced systemic inflammation. In contrast, the scores of sodium, dairy, total protein, and refined grains showed no significant effect. Briefly, our study provides an anti‐inflammatory dietary pattern suggestion based on the 13 components of HEI‐2020 and Dietary Guidelines for Americans.

## Graphical abstract

Weighted quantile regression models and quantile g‐computation models supported the negative association between the mixed components and systemic inflammation and demonstrated that a vegetarian diet especially with high consumption of whole fruits, whole grains, and low consumption of added sugars according to HEI‐2020 benefited against systemic inflammation, while the scores of total protein food and refined grains showed no significant effect.

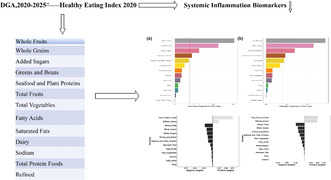

## 1 INTRODUCTION

Inflammation is a multifaceted physiological response that is pivotal for repairing tissue damage, while its chronic form has detrimental effects on tissues and organs (Coussens & Werb, 2002), leading to a variety of health disorders, including aging, obesity, diabetes, arthritis, cancer, cardiovascular diseases, and stroke (Coussens & Werb, 2002; Watson et al., 2017). Recent evidence demonstrates a significant rise in abnormal inflammatory responses and immune system dysfunction, which is associated with an increased prevalence of chronic inflammatory disorders and chronic diseases (Leuti et al., 2020; Qi et al., 2023).

Dietary factors are known to significantly influence the regulation of inflammation and oxidative stress (Aleksandrova et al., 2021; Haß et al., 2019; Koelman et al., 2022). The impact of dietary nutrients on immune and inflammatory responses is an area of research that is gaining traction. A meta‐analysis reveals that the Mediterranean diet and the dietary approaches to stop hypertension (DASH) diet lead to the most substantial reduction in inflammatory biomarkers among several dietary patterns (Aleksandrova et al., 2021), which underscores the role of diet in modulating inflammation (Craddock et al., 2019).

Blood inflammatory indexes, encompassing immune‐inflammatory cells such as lymphocytes and platelets, offer cost‐effective and readily accessible biomarkers for predicting the onset and monitoring the progression of inflammatory diseases (Cheng et al., 2023). Systemic immune‐inflammation index (SII) and systemic inflammation response index (SIRI), derived by platelet and three subtypes of leukocyte, have been proposed to be associated with atherogenesis, cardiovascular diseases, rheumatoid arthritis, depression, and mortality (Cheng et al., 2023; Xia et al., 2023). Several studies demonstrate that SII and SIRI are believed to better reflect the intensity of systemic inflammation than simple white blood cell counts (Fang et al., 2023; Nøst et al., 2021; Yang et al., 2023).

The Healthy Eating Index (HEI) is considered as a gold standard for quantifying dietary quality based on the Dietary Guidelines for Americans (DGA) (“Dietary Guidelines for Americans, 2020–2025”). Studies have consistently linked higher HEI scores with improved health outcomes and a reduced risk of various diseases encompassing periodontitis (Li, Liu et al., 2022), osteoporosis (Fan et al., 2022), and hyperuricemia (Nie et al., 2022). However, the HEI's focus on overall diet quality belies the potential variability in dietary composition among individuals with equivalent scores. This variability underscores the necessity for a nuanced examination of the individual components of the HEI and their specific contributions to health outcomes (Burggraf et al., 2018; Petersen et al., 2022). For example, Nie et al. (2022) reported that dairy products and whole grains contribute most to the decrease in the risk of gout. Sullivan et al. (2021) reported that whole fruits, seafood, and plant proteins are most favorably associated with cardiometabolic health.

In this study, we aim to explore the association of HEI‐2020 and its components with systemic inflammatory biomarkers within a representative sample of the US adult population. Furthermore, the independent and joint effects of 13 components on systemic inflammatory biomarkers are evaluated by fitting generalized linear models, weighted quantile regression (WQS) models, and quantile g‐computation (QGC) models. Our research hypothesis posits that a higher HEI‐2020 score is inversely related to both SII and SIRI, suggesting that certain components of HEI‐2020 may exert a significant influence on these inflammatory markers, while others may not contribute as substantially. The results of this study help us understand which foods and nutrients contribute most to mitigate systemic inflammation.

## 2 MATERIALS AND METHODS

### 2.1 Study population

The National Health and Nutrition Examination Survey (NHANES) is an epidemiological program aimed to assess the health and nutritional conditions of individuals aged ≥2 years in the United States. The program protocol is approved by the Institutional Review Board of the Centers for Disease Control and Prevention (CDC) National Center for Health Statistics (https://www.cdc.gov/nchs/nhanes/index.htm).

**FIGURE 1:**
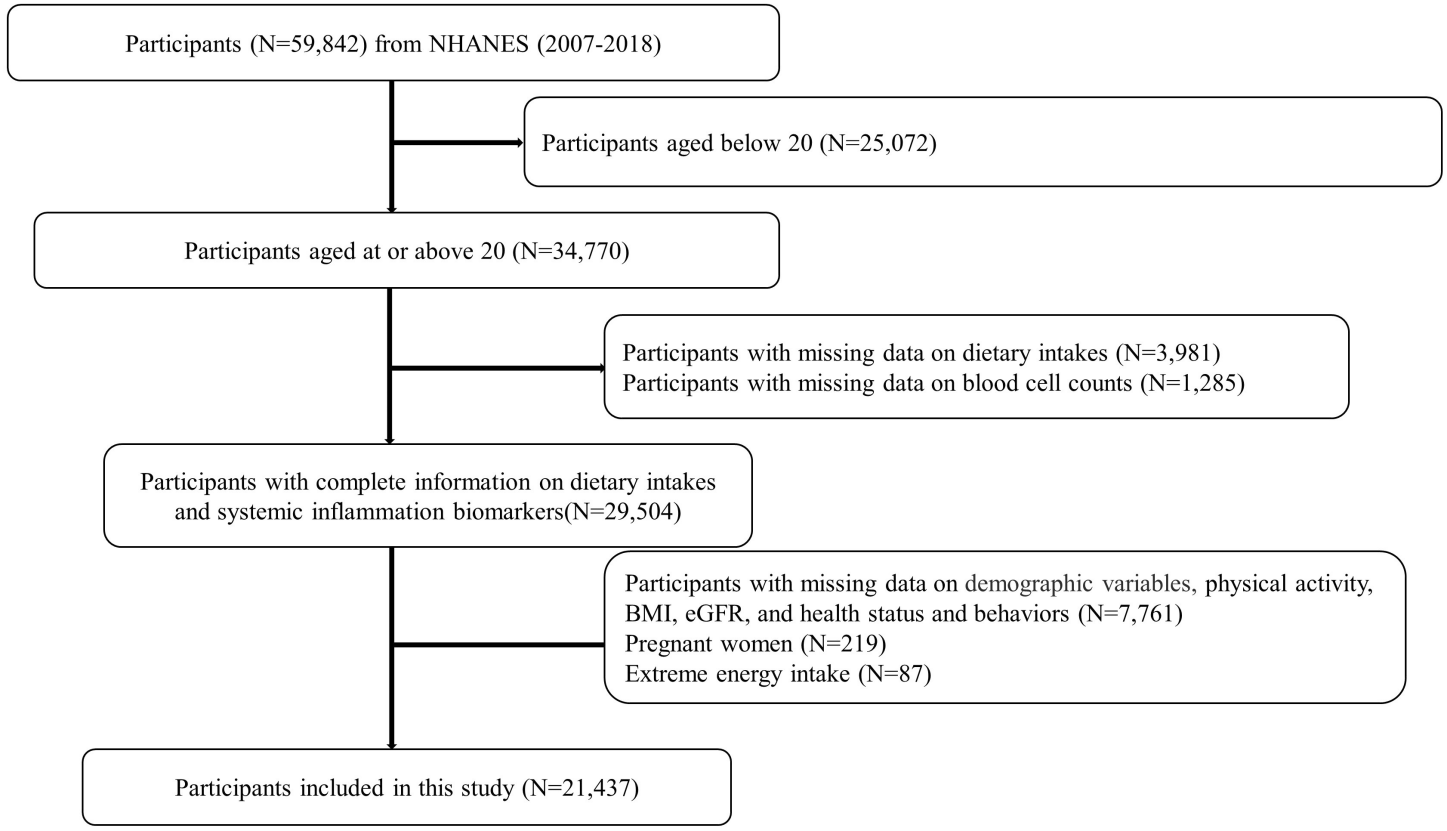
Flowchart of sample selection.

This study included NHANES datasets from 2007 to 2018. Among 59,842 participants from NHANES 2007–2018, we excluded 25,072 participants aged below 20, 3981 participants with missing data on dietary intake information, 1285 participants with missing data on blood cell count information, 7761 participants with missing data on demographic variables, physical activity, BMI, eGFR, and health status and behaviors, 219 pregnant women, and 87 participants with extreme energy intake levels (men: >8000 or <500 kcal/day; women: >5000 or <500 kcal/day). Finally, a total of 21,437 participants were finally recruited in the study. Figure 1 presented the details of the study design, sampling, and exclusion.

### 2.2 Measurement of HEI‐2020

Dietary intake data were collected by 24‐h dietary recalls and participants who had two complete 24‐h dietary recalls that were reliable and met the minimum criteria were included. HEI‐2020, the latest version of HEI, was utilized to assess the dietary quality, which consisted of the same components as HEI‐2015 (Krebs‐Smith et al., 2018; Shams‐White et al., 2023). The components are grouped into two forms, nine adequacy components (total fruits, whole fruits, total vegetables, greens and beans, whole grains, dairy, total protein foods, seafood and plant proteins, and fatty acids) and four moderation components (refined grains, sodium, added sugars, and saturated fats) (Krebs‐Smith et al., 2018). Total fruits, whole fruits, total vegetables, greens and beans, total protein foods, and seafood and plant proteins have a maximum possible score of 5, while the other seven components have a maximum possible score of 10. HEI‐2020 scores range from 0 to 100, with higher HEI scores reflecting higher diet quality. The details of the HEI‐2020 and its components have been described elsewhere (Shams‐White et al., 2023).

### 2.3 Definitions of systemic inflammatory biomarkers

Two systemic inflammatory biomarkers are calculated, with complete data on lymphocyte, neutrophil, monocyte, and platelet counts. Blood cell count measurements of blood specimens are detected on mobile examination center (MEC) automated analytical instrument (Beckman Coulter MAXM; Beckman Coulter Inc.). Details could be acquired at https://wwwn.cdc.gov/Nchs/Nhanes/2015‐2016/CBC_I.htm.

SII and SIRI are calculated through the following formulas (Hu et al., 2014; Qi et al., 2016):

$$\mathrm{SII}=\left(\text{platelet count}\times \text{neutrophil count}\right)/\text{lymphocyte count};$$

$$\text{SIRI}=\left(\text{neutrophil count}\times \text{monocyte count}\right)/\text{lymphocyte count}.$$

### 2.4 Other variables of interest

Similar to previous studies, covariates of interest consisted of sociodemographic, behavioral, and health characteristics.

Socioeconomic and demographic factors were categorized as follows: Age was divided into three brackets: 20–39, 40–59, and 60 years and older. Sex was recorded as either female or male. Race was detailed into Hispanic (including Mexican American and other Hispanic groups), Non‐Hispanic White, Non‐Hispanic Black, and Other/multiracial. Education levels were classified into three categories: less than a high school diploma, a high school diploma or GED, and some college education or higher. The family income‐to‐poverty ratio (FIPR) was stratified into three ranges: <1.30, between 1.30 and 3.50, and >3.50 (Montgomery et al., 2017).

Behavioral characteristics comprised smoke exposure, alcohol drinking, physical activity, and total energy intake per day. Smoke exposure was assessed by the serum cotinine levels (<0.015 ng/mL; ≥0.015 ng/mL) (Tao et al., 2021). Alcohol drinking was classified into two groups: no (those who had less than 12 alcohol drinks per year) and yes (those who had at least 12 alcohol drinks per year) (Hicks et al., 2021). The physical activity was assessed by the Global Physical Activity Questionnaire (GPAQ), which included questions related to daily activities, leisure time activities, and sedentary activities further categorized by metabolic equivalent (MET) scores. Finally, physical activity levels were classified into three groups: no physical activity group (0 MET‐h/week), low‐intensity physical activity group (≤48 MET‐h/week), and high‐intensity physical activity group (>48 MET‐h/week) (Tian et al., 2022). The total energy intake per day was the average of two 24‐h dietary recalls.

Health factors included body mass index (BMI) levels, hypertension, diabetes, and estimated glomerular filtration rate (eGFR). BMI was calculated by dividing weight by square of height (kg/m^2^) and divided into three stratifications: ≤24.9, 25–29.9, and >30 kg/m^2^ (Dreimüller et al., 2019). Diabetes was defined as self‐reported diabetes diagnosed by a doctor or health professional, glycohemoglobin ≥6.5%, fasting glucose ≥7 mmol/L, 2‐h glucose of oral glucose tolerance test, or serum glucose≥11.1 mmol/L (Xie et al., 2020). Hypertension was defined as self‐reported hypertension diagnosed by a doctor or health professional, and/or anti‐hypertension medication, and/or systolic blood pressure ≥130 mmHg and/or diastolic blood pressure ≥80 mmHg (Whelton et al., 2018). The CKD‐Epidemiology Collaboration (CKD‐EPI) equation was adopted to calculate eGFR (Levey et al., 2009): eGFR = 141 × min (Scr/κ, 1)^α^ × max (Scr/κ, 1)^−1.209^ × 0.993^Age^ × 1.018 [if female] × 1.159 [if black], where Scr denotes serum creatinine, κ is 0.9 for male and 0.7 for female, α is −0.411 for male and −0.329 for female, min represents the minimum of Scr/κ or 1, and max expresses the maximum of Scr/κ or 1.

### 2.5 Statistical analyses

Given the complex sampling survey, an appropriate weight (MEC weight) was adopted. Continuous variables were presented as weighted mean and standard deviation (SD) or median and interquartile range (IQR). Categorical variables were presented as frequency and weighted proportion. The data for SII and SIRI were transformed using the natural logarithm due to the skewed concentrations, and the results of this transformation were applied to subsequent analyses.

Weighted generalized linear model was used to evaluate the linear relationship between HEI‐2020 (including continuous variables and quantile groups) and Ln‐SII and Ln‐SIRI. Model 1 was a crude model. Model 2 was adjusted for age, sex, and race. Model 3 was further adjusted for FIPR, educational levels, BMI levels, alcohol drinking, serum cotinine levels, physical activity, total energy intake, eGFR, diabetes, and hypertension. The restricted cubic spline (RCS) analyses were conducted to explore the dose–response relationships between HEI‐2020 and Ln‐SII and Ln‐SIRI. Multiple weighted generalized linear models were used to analyze the linear relationship between 13 independent HEI‐2020 components and Ln‐SII and Ln‐SIRI, with adjusting for age, sex, and race, FIPR, educational levels, BMI levels, drinking status, serum cotinine levels, physical activity, total energy intake, eGFR, diabetes, and hypertension. Then the false discovery rate (FDR) correction was conducted to correct multiple tests for *p*‐values.

The WQS regression model was originally designed to evaluate the mixed effect of environmental pollutants on the outcome by building weighted combinatorial quantiles (Carrico et al., 2015). Recently, some studies used WQS regression models to evaluate the mixed effect of complex dietary intakes (Chen et al., 2023). To estimate the parameters, 40% of data were randomly divided into a training data set, and 60% of them were assigned to a validation data set, with 1000 bootstrapped samples used. The effects of all components were forcibly restricted to the same direction in the WQS model, and the total weights of all components were summed to 1.

Quantile g‐computation was a technique for evaluating the synergistic effects of multiple chemicals that combine the simplicity of WQS regression with the flexibility of g‐computation to permit inference on unbiased mixture effects (Keil et al., 2020). The positive or negative weights of the mixed components were summed to 1. Here, we performed QGC models to evaluate the mixed effect of the components of HEI‐2020.

For all measures, the 95% confidence intervals (CI) were obtained. The statistical analyses were conducted using R software (version 4.1.2) and a two‐tailed *p*‐value <.05 was considered statistically significant.

## 3 RESULTS

### 3.1 Characteristics of study participants

Table 1 presented the characteristics of study participants. Among the participants, 33.8% of the weighted sample were aged 20–39 years, 38.3% were aged 40–59, and 27.9% were aged 60–80. More than half of the weighted participants were women (51.3%), non‐Hispanic White (70.7%), consumed alcohol (77.7%), and had serum cotinine no lower than 0.015 ng/mL (65.6%). Among the weighted participants, 14.1% had diabetes and 45.9% had hypertension. The weighted mean of HEI‐2020 was 53.95 ± 13.45. The weighted median and IQR of total energy intake, SII, and SIRI were 1982.00 (1938.50, 2543.00), 463.05 (336.52, 645.58), and 1.05 (0.73, 1.53), respectively.

**TABLE 1:** Characteristics of the study participants.

Variables	Overall (*n* = 21,437)
*Age*, *years*, *n* (*weighted %*)	
20 ~ 39	6766 (33.8)
40 ~ 59	7182 (38.3)
60 ~ 80	7489 (27.9)
*Sex*, *n* (*weighted %*)	
Men	10,501 (48.7)
Women	10,936 (51.3)
*Race* (*%*)	
Hispanic	5118 (12.8)
Non‐Hispanic White	9780 (70.7)
Non‐Hispanic Black	4296 (9.8)
Other or multiracial	2243 (6.7)
*FIPR*, *n* (*weighted %*)	
≤1.30	6447 (19.8)
1.30–3.50	8189 (35.7)
>3.50	6801 (44.5)
*Educational levels*, *n* (*weighted %*)	
Below high school	4576 (13.7)
High school	4923 (22.9)
Some college or AA degree	6561 (31.7)
College graduate or above	5377 (31.7)
*Alcohol drinking*, *n* (*weighted %*)	
Yes	15,504 (77.7)
No	5933 (22.3)
*Serum cotinine*, *ng/mL*, *n* (*weighted %*)	
<0.015	6535(34.4)
≥0.015	14,902 (65.6)
Diabetes, *n* (weighted %)	4039 (14.1)
Hypertension, *n* (weighted %)	10,871 (45.9)
*BMI*, *kg/m* ^*2*^, *n* (*weighted %*)	
<25	5871 (28.5)
25 to <30	7043 (32.8)
≥30	8523 (38.7)
*Physical activity*, *n* (*weighted %*)	
No	5475 (21.2)
Low	9319 (45.8)
High	6643 (33.0)
eGFR	93.20 (21.82)
HEI‐2020	53.95 (13.45)
Total energy intake, kcal/day	1982.00 [1538.50, 2543.00]
SII	463.05 [336.52, 645.58]
SIRI	1.05 [0.73, 1.53]

Abbreviations: BMI, body mass index; eGFR, estimated glomerular filtration rate; FIPR, family income‐to‐poverty ratio; HEI‐2020, Healthy Eating Index 2020; SII, systemic immune‐inflammation index; SIRI, systemic inflammation response index.

### 3.2 Association between HEI‐2020 and systemic inflammatory biomarkers

Association between HEI‐2020 and systemic inflammatory biomarkers was presented in Table 2. After adjustment for all covariates, for each unit increase in the HEI‐2020, the Ln‐SII was estimated to decrease by 0.0025. For each unit increase in the HEI‐2020, the Ln‐SIRI was estimated to decrease by 0.0022. Compared with the first quantile, the multivariate‐adjusted β (quartile, 95% CI) of Ln‐SII were −0.0242 (Q2, −0.0471 to −0.0014), −0.0506 (Q3, −0.0726 to −0.0287), and −0.0841 (Q4, −0.1097 to −0.0586). Compared with the first quantile, the multivariate‐adjusted β (quartile, 95% CI) of Ln‐SIRI were −0.0497 (Q3, −0.0794 to −0.0200) and −0.0666 (Q4, −0.0937 to −0.0395).

**TABLE 2:** Associations between HEI‐2020 and systemic inflammatory biomarkers.

	Model 1, β (95% CI)	Model 2, β (95% CI)	Model 3, β (95% CI)
*SII*			
HEI‐2020 (continuous)	−0.0025 (−0.0031, −0.0020)	−0.0035 (−0.0040, −0.0029)	−0.0025 (−0.0032, −0.0019)
Q1	Ref.	Ref.	Ref.
Q2	−0.0220 (−0.0448, 0.0008)	−0.0313 (−0.0539, −0.0088)	−0.0242 (−0.0471, −0.0014)
Q3	−0.0479 (−0.0700, −0.0257)	−0.0680 (−0.0898, −0.0462)	−0.0506 (−0.0726, −0.0287)
Q4	−0.0857 (−0.1092, −0.0622)	−0.1168 (−0.1401, −0.0934)	−0.0841 (−0.1097, −0.0586)
*p* for trend	<.001	<.001	<.001
*SIRI*			
HEI‐2020 (continuous)	−0.003 (−0.0037, −0.0023)	−0.0038 (−0.0045, −0.0031)	−0.0022 (−0.0029, −0.0015)
Q1	Ref.	Ref.	Ref.
Q2	−0.0204 (−0.0483, 0.0075)	−0.0329 (−0.0599, −0.0058)	−0.0196 (−0.0467, 0.0074)
Q3	−0.0623 (−0.0940, −0.0307)	−0.0809 (−0.1114, −0.0503)	−0.0497 (−0.0794, −0.0200)
Q4	−0.0936 (−0.1216, −0.0657)	−0.1240 (−0.1507, −0.0973)	−0.0666 (−0.0937, −0.0395)
*p* for trend	<.001	<.001	<.001

*Note*: Model 1 was a crude model without adjustment. Model 2 was adjusted for age, sex, and race. Model 3 was further adjusted for FIPR, educational levels, BMI levels, drinking status, serum cotinine levels, physical activity, total energy intake, eGFR, diabetes, and hypertension status.

Abbreviations: CI, confidence intervals; HEI‐2020, Healthy Eating Index 2020; SII, systemic immune‐inflammation index; SIRI, systemic inflammation response index.

**FIGURE 2:**
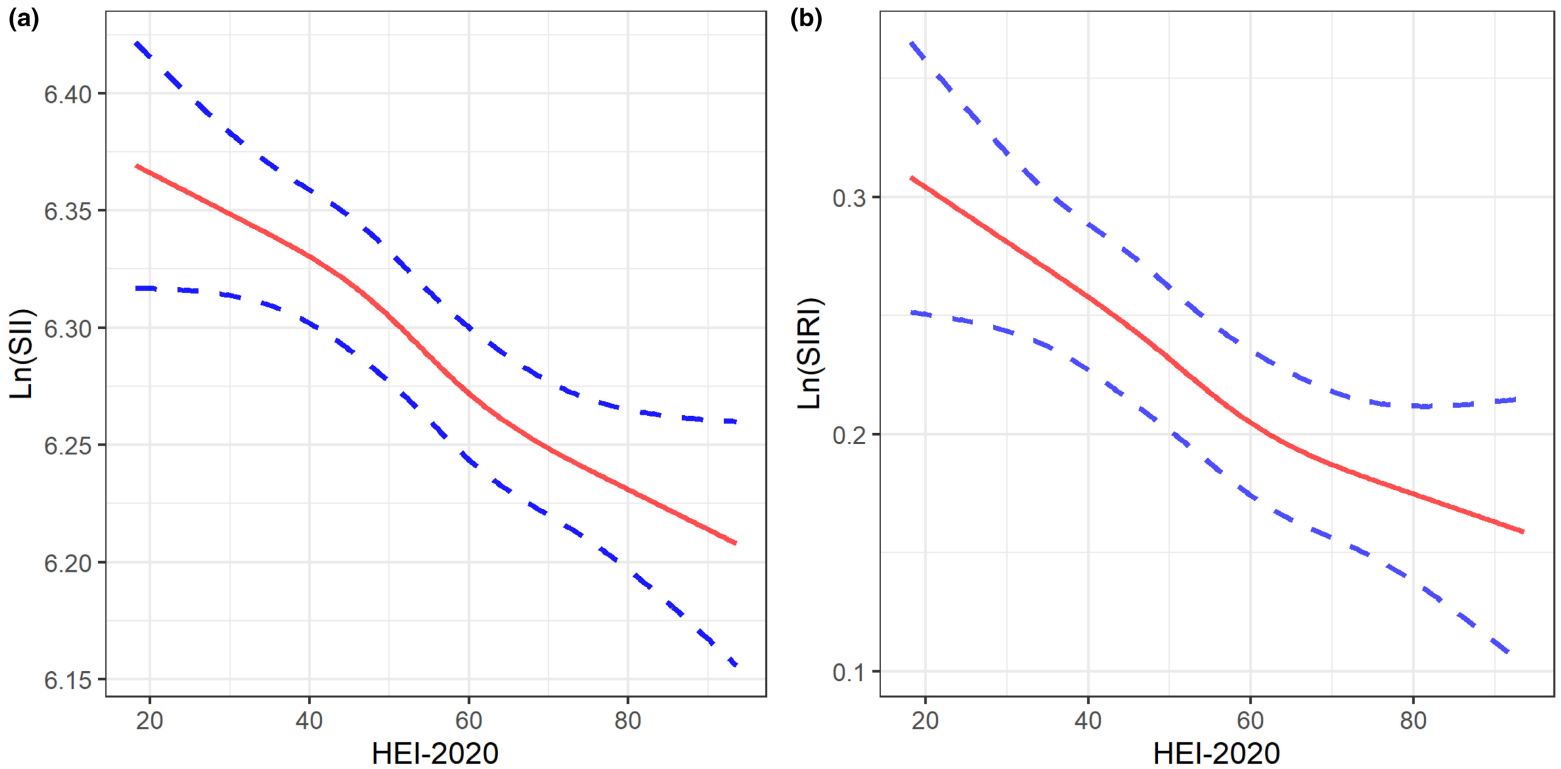
Dose–response relationships between HEI‐2020 and Ln‐SII (a) and Ln‐SIRI (b).

The dose–response relationships between HEI‐2020 and Ln‐SII and Ln‐SIRI are presented in Figure 2, which also supported the linear associations.

### 3.3 Association between 13 components and systemic inflammatory biomarkers

The independent effect of all components was presented in Table 3. The multivariate linear regression models revealed that scores of whole fruits, whole grains, added sugars, greens and beans, seafood and plant protein, and total fruits were inversely associated with both Ln‐SII and Ln‐SIRI. FDR corrections were conducted for multiple tests and confirmed the significant associations, while the scores of refined grains, dairy, total protein food, and sodium showed no significant association with both Ln‐SII and Ln‐SIRI.

**TABLE 3:** Independent effect of HEI‐2020 components on systemic inflammatory biomarkers.

Component	SII			SIRI		
	β (95% CI)	*p*	P‐FDR	β (95% CI)	*p*	P‐FDR
Total vegetables	−0.0069 (−0.0137,0.0000)	.050	0.072	−0.0121 (−0.0189, −0.0054)	<.001	0.001
Greens and beans	−0.0081 (−0.0121, −0.0041)	<.001	<0.001	−0.0105 (−0.0149, −0.0062)	<.001	<0.001
Total fruits	−0.0104 (−0.0153, −0.0055)	<.001	<0.001	−0.0094 (−0.0146, −0.0042)	<.001	0.001
Whole fruits	−0.0132 (−0.0174, −0.0090)	<.001	<0.001	−0.0124 (−0.0168, −0.0081)	<.001	<0.001
Whole grains	−0.0089 (−0.0119, −0.0059)	<.001	<0.001	−0.0075 (−0.0107, −0.0044)	<.001	<0.001
Dairy	−0.0007 (−0.0039, 0.0025)	.650	0.704	0.0004 (−0.0033, 0.0041)	.826	0.826
Total protein foods	−0.0032 (−0.0123, 0.0060)	.494	0.584	0.0014 (−0.0091, 0.0119)	.790	0.826
Seafood and plant proteins	−0.0079 (−0.0115, −0.0043)	<.001	<0.001	−0.0082 (−0.0126, −0.0039)	<.001	<0.001
Fatty acids	−0.0032 (−0.0062, −0.0002)	.038	0.061	−0.0053 (−0.0081, −0.0026)	<.001	<0.001
Sodium	0.0005 (−0.0025, 0.0034)	.751	0.751	0.0017 (−0.0013, 0.0047)	.272	0.321
Refined grains	0.0012 (−0.0013, 0.0038)	.332	0.431	0.0029 (0.0001, 0.0057)	.043	0.062
Saturated fats	−0.0035 (−0.0066, −0.0005)	.022	0.041	−0.0026 (−0.0057, 0.0006)	.105	0.136
Added sugars	−0.0089 (−0.0119, −0.0059)	<.001	<0.001	−0.0062 (−0.0097, −0.0026)	.001	0.001

*Note*: Models were adjusted for age, sex, race, FIPR, educational levels, BMI levels, drinking status, serum cotinine levels, physical activity, total energy intake, eGFR, diabetes, and hypertension status.

Abbreviations: CI, confidence intervals; SII, systemic immune‐inflammation index; SIRI, systemic inflammation response index.

### 3.4 Association between combined effect of HEI‐2020 components with systemic inflammatory biomarkers

**FIGURE 3:**
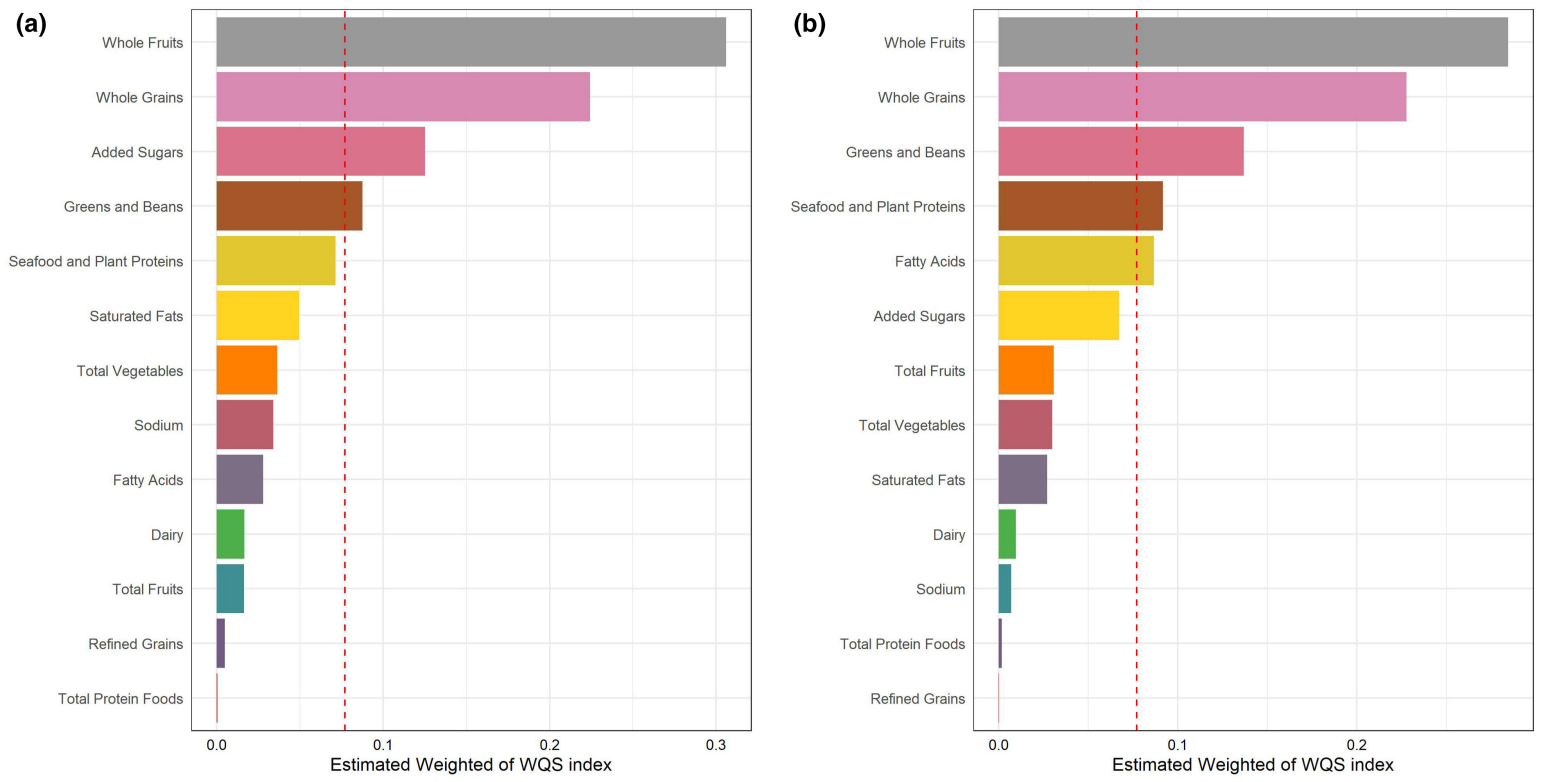
The weights of each component in negative weighted quantile regression model regression index for markers of systemic inflammation. (a) SII; (b) SIRI.

We performed the WQS regression model and QGC model to investigate the potential effects of mixed components of HEI‐2020 (Table 4). The WQS indices were significantly negatively correlated with the SII and SIRI. Specifically, the WQS models revealed negative associations between the mixed components of HEI‐2020 and Ln‐SII (WQS β: −0.0992; 95% CI: −0.1244 to −0.0739, *p* < .001) and Ln‐SIRI (WQS β: −0.1062; 95% CI: −0.1330 to −0.0794, *p* < .001). The details of weights obtained by WQS regression models were shown in Table S1 and Figure 3. The highest weighted component for Ln‐SII was whole fruits, followed by whole grains, seafood and plant proteins, and added sugars. The highest weighted component for Ln‐SIRI was whole fruits, followed by whole grains, greens, and beans.

**TABLE 4:** Association between mixture components of HEI‐2020 with systemic inflammatory biomarkers by weighted quantile regression (WQS) and quantile g‐computation (QGC) analysis.

Model	Outcomes	β	95% CI	*p*	Direction
WQS	SII	−0.0992	−0.1244, −0.0739	<.001	Negative
		Not estimated	NA	NA	Positive
	SIRI	−0.1062	−0.1330, −0.0794	<.001	Negative
		Not estimated	NA	NA	Positive
QGC	SII	−0.1005	−0.1308, −0.0727	<.001	Negative
	SIRI	−0.1016	−0.1340, −0.0691	<.001	Negative

*Note*: Models were adjusted for age, sex, and race, FIPR, educational levels, BMI levels, drinking status, serum cotinine levels, physical activity, total energy intake, eGFR, diabetes, and hypertension status.

Abbreviations were the same as in Table 3.

**FIGURE 4:**
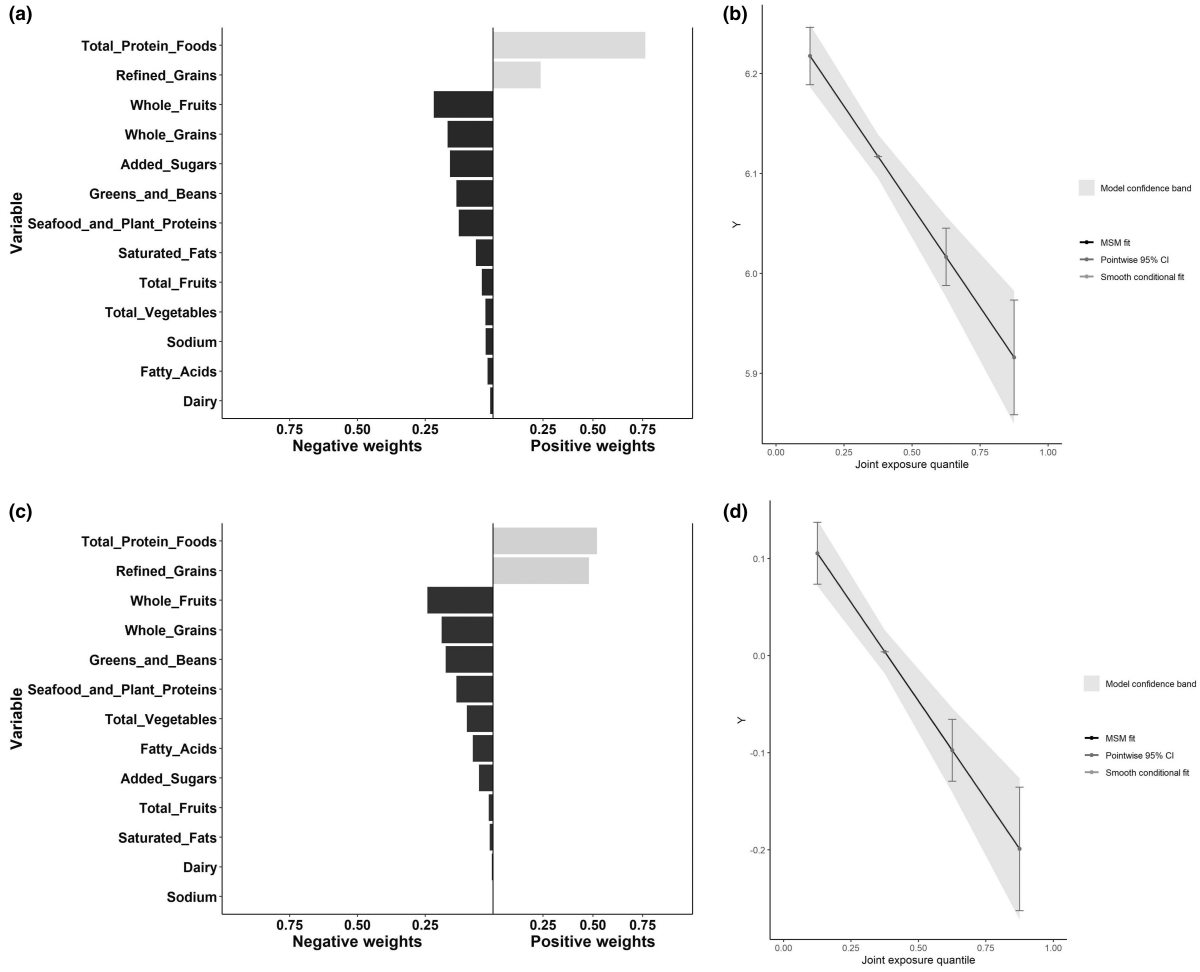
The proportion of positive and negative effects of mixed components on markers of systemic inflammation and the combined effects in quantile g‐computation model. (a) Proportion for SII; (b) Combined effects for SII; (c) Proportion for SIRI; (d) Combined effects for SIRI.

Similar results were obtained in QGC models, which revealed negative association between the mixed components of HEI‐2020 and Ln‐SII (β: −0.1005; 95% CI: −0.1308 to −0.0727, *p* < .001) and Ln‐SIRI (β: −0.1016; 95% CI: −0.1340 to −0.0691, *p* < .001). And the details of weights obtained by QGC models are shown in Table S2 and Figure 4. The highest negative weighted component for Ln‐SII was whole fruits, followed by whole grains, and added sugars. The highest negative weighted component for Ln‐SIRI was whole fruits, followed by whole grains, greens and beans, and seafood and plant proteins.

## 4 DISCUSSION

In the present study, we assessed the associations of HEI‐2020 and its 13 components with systemic inflammatory biomarkers, SII and SIRI. Furthermore, WQS regression models and QGC models were used to estimate the combined effect of complex components and their contributing weights for systemic inflammation, which suggested that whole fruits and whole grains had the highest contributive effect on SII and SIRI reduction. These findings suggested that a high‐quality diet following the DGA, with high consumption of vegetarian food (especially whole fruits and whole grains) and low intake of added sugars, may help regulate these biomarkers and potentially benefit the immune system.

Previous studies have predominantly focused on the relationship between the overall dietary patterns and inflammation (Aleksandrova et al., 2021; Koelman et al., 2022). Generally, current evidence supported that high‐quality dietary patterns help reduce inflammation (Aleksandrova et al., 2021; Koelman et al., 2022). Comprehensive evidence showed that adherence to a Mediterranean‐type diet or a DASH diet led to a reduction in several inflammatory biomarkers, including interleukin‐6 (IL‐6), IL‐1β, high‐sensitivity C‐reactive protein (hs‐CRP), IL‐8, and tumor necrosis factor α (TNF‐α) (Koelman et al., 2022; Luisi et al., 2019; Whalen et al., 2016), whereas a positive association was found for western and fast food diets (Aleksandrova et al., 2021; Christ et al., 2019). One potential explanation for this discrepancy lies in the significant variations observed in the food components that characterize these dietary patterns. Briefly, a Mediterranean diet consists of high consumption of fruits, whole grains, vegetables, legumes, and olive oil, moderate‐to‐high intake of fish and seafood, and low consumption of meat products, dairy and sweets (Widmer et al., 2015), while western/fast food dietary pattern is characterized by high levels of fat and sugars and low intake of greens (Christ et al., 2019; Molino et al., 2022). The HEI is used for quantifying the consistency between the actual dietary consumption of Americans and the recommendations of the DGA, rather than just evaluating dietary patterns. Vahidet al. reported a significant inverse correlation between HEI‐2015 and inflammatory biomarkers, like IL‐4 (*R* = −0.063), IL‐1β (*R* = −0.054), and hs‐CRP (*R* = −0.069) (Vahid et al., 2022). Similarly, our study demonstrated that total HEI‐2020 scores and the combined effect of 13 components were inversely associated with SII and SIRI.

Specific components of the HEI have been linked to health benefits. Specifically, higher scores for total vegetables, greens and beans, total fruits, whole fruits, and whole grains were associated with a lower risk of osteoporosis in elderly adults (Fan et al., 2022). Additionally, higher scores for whole fruits and seafood and plant proteins were linked to better cardiometabolic health (Sullivan et al., 2021). Therefore, in our study, we gave particular attention to the role and contribution of the 13 independent components of HEI‐2020 to systemic inflammation. The WQS and QGC models estimated the potential contribution weights of the 13 components for Ln‐SII and Ln‐SIRI. Despite variations in the ranking of the components' influence across models, whole fruits and whole grains consistently emerged as the most influential factors. In addition, added sugars, green vegetables and beans, seafood and plant‐based proteins, and total fruits were identified as significant contributors to the mitigation of systemic inflammation, while dairy, sodium, refined grains, and total protein foods did not demonstrate a statistically significant association. Although the anti‐inflammatory components we identified shared similarities with those of the Mediterranean and DASH diets, there were additional nuances that warranted a more thorough exploration.

In our study, whole fruits and whole grains were two most important components that reduced inflammation. They are rich in fiber, phytochemicals, antioxidants like vitamins, and minerals, are known to attenuate inflammation, and reduce the risk of inflammation‐associated diseases (Dreher, 2018; Liu, 2013; Sang et al., 2020). Their direct anti‐inflammatory properties, as well as those modulated by gut microbiota, have been robustly validated across a spectrum of human and animal research studies (De Filippis et al., 2016; Dreher, 2018; Martínez et al., 2013; Roager et al., 2019). Green vegetables are potent in mitigating inflammation due to their rich content of antioxidant nutrients (Li, Zhang et al., 2022). Soybeans and seafood are the primary sources of essential omega‐3 fatty acids, which have been shown to reduce inflammation (Djuricic & Calder, 2021; Fan et al., 2022; Islam et al., 2023). In addition, the present study showed that lower intake of added sugars was associated with lower inflammation because added sugars were scored in inverse (Shams‐White et al., 2023). Previous studies reported that added sugar consumption was likely to trigger a hyperinflammatory and overoxidized state (Lula et al., 2014). In line with our results, a meta‐analysis presented a thorough examination of the existing data, which indicated that there was insufficient evidence to confirm the anti‐inflammatory effects of dairy products (Hess et al., 2021).

Our study had some strengths. First, we used a large, nationally representative sample from NHANES. Data from two 24‐h dietary reliable recalls were used to avoid recall bias. Second, we evaluated the combined effects of 13 components of HEI‐2020 by WQS regression models and QGC models, which have recently been used in nutrition‐related studies. The results identified the highest contributing dietary components for systemic inflammatory biomarkers. These methods provided a new insight into the effects of dietary intake recommendations on specific health outcomes. Third, two novel inflammation markers, SII and SIRI, calculated by blood cell count index, were used to reflect systemic inflammatory levels, which can also reflect systemic immunity levels.

Nevertheless, some limitations in our study should be noticed. The cross‐sectional study design cannot establish a causal relationship. And all the participants were from NHANES program, which limited the generalization of our results. Furthermore, despite some potential covariates were adjusted in our analyses, we were incapable of totally ruling out the impact of additional potential confounding variables, including living environment, metabolites, and genetic structures.

## 5 CONCLUSIONS

In conclusion, our results revealed a negative linear relationship between HEI‐2020 and two systemic inflammatory biomarkers, SII and SIRI. High intakes of whole fruits, whole grains, greens and beans, and seafood and plant proteins, along with a low intake of added sugars, were associated with reduced systemic inflammation. The findings may be helpful to guide the nutritional intake for controlling inflammation.

## AUTHOR CONTRIBUTIONS

**Hongguang Yang:** Conceptualization (equal); formal analysis (lead); methodology (lead); writing – original draft (lead); writing – review and editing (lead). **Yao Liu:** Formal analysis (equal); software (equal); validation (equal); writing – original draft (equal); writing – review and editing (equal). **Zhenhe Huang:** Formal analysis (equal); writing – original draft (equal). **Guifang Deng:** Conceptualization (equal); funding acquisition (equal); software (equal); writing – original draft (equal); writing – review and editing (equal).

## FUNDING INFORMATION

This work was supported by grants from the National Natural Science Foundation of China (grant number 82103821), the Shenzhen Science and Technology Innovation Committee (grant number JCYJ20210324112400002), the Major Technological Project of Shenzhen Nanshan District Health System (grant number NSZD2023024), and the Foundation of Guangdong Provincial Key Laboratory of Nutritional Diet and Health (grant number 2022‐GLFNH‐04).

## CONFLICT OF INTEREST STATEMENT

The authors declared that no competing interest existed.

## ETHICS STATEMENT

The data come from NHANES. No Ethics approval and consent to participate is needed.

## Supporting information


Table S1.

## Data Availability

Data used in this study are available on the NHANES website (https://www.cdc.gov/nchs/nhanes/index.htm).
