# Supplementary material for: The Healthy Eating Index 2020 components contributed unequally to systemic inflammatory biomarkers: A large national cross‐sectional study
Source: Food Sci Nutr. 2024 Jul 16;12(10):7212–22. doi: 10.1002/fsn3.4347 (PMC11521706; doi:10.1002/fsn3.4347)
Supplement: Supplementary file 1 — Table S1. [file FSN3-12-7212-s001.docx]

# Supplementary materials

**Table S1. weights of the components in WQS models**

| Components | SII | SIRI |
| --- | --- | --- |
| Total Vegetables | 9.04% | 7.49% |
| Total Fruits | 3.59% | 5.77% |
| Whole Fruits | 20.70% | 25.30% |
| Whole Grains | 15.50% | 11.50% |
| Dairy | 2.59% | 0.39% |
| Total Protein Foods | 0.28% | 0.74% |
| Seafood and Plant Proteins | 14.57% | 10.20% |
| Fatty Acids | 0.65% | 8.99% |
| Sodium | 7.45% | 0.19% |
| Refined Grains | 0.02% | 0.02% |
| Saturated Fats | 7.94% | 1.40% |
| Added Sugars | 13.90% | 9.21% |
| Greens and Beans | 3.77% | 18.80% |

Abbreviations: SII, systemic immune-inflammation index; SIRI, systemic inflammation response index

**Table S2. weights of the components in QGC models**

| Component | SII | |  | SIRI | |
| --- | --- | --- | --- | --- | --- |
|  | Negative weights | Positive weights |  | Negative weights | Positive weights |
| Total Vegetables | 9.68% |  |  | 2.89% |  |
| Total Fruits | 1.67% |  |  | 4.12% |  |
| Whole Fruits | 24.22% |  |  | 21.86% |  |
| Whole Grains | 18.92% |  |  | 16.81% |  |
| Dairy | 0.46% |  |  | 1.06% |  |
| Total Protein Foods |  | 52.00% |  |  | 76.20% |
| Seafood and Plant Proteins | 13.61% |  |  | 12.66% |  |
| Fatty Acids | 7.49% |  |  | 2.03% |  |
| Sodium | 0.01% |  |  | 2.77% |  |
| Refined Grains |  | 48.00% |  |  | 23.80% |
| Saturated Fats | 1.24% |  |  | 6.32% |  |
| Added Sugars | 5.19% |  |  | 15.95% |  |
| Greens and Beans | 17.51% |  |  | 13.53% |  |
| Sum of coefficients | -0.130 | 0.028 |  | -0.120 | 0.019 |

Abbreviations: SII, systemic immune-inflammation index; SIRI, systemic inflammation response index
